# The trend of caesarean birth rate changes in China after ‘universal two-child policy’ era: a population-based study in 2013–2018

**DOI:** 10.1186/s12916-020-01714-7

**Published:** 2020-09-15

**Authors:** Jie Yan, Long Wang, Ying Yang, Ya Zhang, Hongguang Zhang, Yuan He, Zuoqi Peng, Yuanyuan Wang, Qiaomei Wang, Haiping Shen, Yiping Zhang, Donghai Yan, Xu Ma, Huixia Yang

**Affiliations:** 1 Department of Obstetrics and Gynecology, Peking University First Hospital, No.1 Xi’anmen Street, Xicheng District, Beijing, 100034 China; 2 China DOHaD Research Centre, China DOHaD Research Centre, National Human Genetic Resources Centre, No. 12 Dahuisi Road, Haidian District, Beijing, 100081 China; 3 National Research Institute for Family Planning, No. 12 Dahuisi Road, Haidian District, Beijing, 100081 China; 4 School of Public Health, Lanzhou University, No. 199 Donggang Road (West), Chengguang District, Lanzhou, 730000 China; 5 Graduate School of Peking Union Medical College, No. 9 Dong Dan San Tiao, Dongcheng District, Beijing, 100005 China; 6 Department of Maternal and Child Health, National Health Commission of the PRC, No. 1 Xizhimenwai Road (South), Xicheng District, Beijing, 100044 China

**Keywords:** Caesarean birth rate, Universal two-child policy, National Free Pre-Pregnancy Check-ups Project (NFPCP)

## Abstract

**Background:**

The universal two-child policy in China which was announced in October 2015 might affect the caesarean birth rate. Few studies reported the caesarean birth rate affected by the policy especially after the universal two-child policy period. This study aimed to demonstrate the caesarean birth rate changes before and after the universal two-child policy and further elaborate the underlying influencing factors.

**Methods:**

This nationwide, retrospective, population-based study was based on National Free Pre-Pregnancy Check-ups Project (NFPCP). Couples planning to conceive in next 6 months were encouraged to participate in NFPCP. Baseline information was collected using a standardized questionnaire with a face-to-face interview, and medical examinations were conducted. Two rounds of follow-up interviews were then conducted by trained nurses to update pregnancy status and outcomes. NFPCP participants who completed deliveries before December 31, 2018, were included in the current study. We used marginal effect of year to examine the trend of caesarean birth rate over time and interrupted time series (ITS) analysis to determine impacts of the universal two-child policy on the trend of caesarean birth rate.

**Results:**

A total of 9,398,045 participants were included in the final analysis. High-risk factors to increase caesarean birth rate were identified. In the current study, the standardized caesarean birth rate declined from 34.1% in 2013 to 31.8% in 2015 and increased to 35.6% in 2018. ITS analysis showed the caesarean birth rate decreased by 0.1% (95% CI 0.1–0.1) per month before the release of universal two-child policy, 1.3% (95% CI 0.6–2.1%) absolute drop during the policy release month, and increased by 0.2% (95% CI 0.1–0.2%) per month after the policy implementation. For the period after the policy release, the increasing trends were observed in rural participants and urban primiparas. The prevalence of caesarean birth rates within China varied regionally.

**Conclusions:**

The decreasing trend of caesarean birth rate was reported after immediate release of the universal two-child policy. An increasing trend of caesarean birth rate was observed 2–3 years after the policy. It reminds us that the caesarean birth rate control is a long-lasting process and all the strategies need to be continually reinforced.

## Background

Caesarean birth is a surgery with both short- and long-term risks compared to vaginal birth, such as surgical complications, neonatal intensive care unit admission, and higher costs [1–3]. Globally, there are disparities between countries regarding caesarean birth rate. The recent data showed caesarean birth rates were more than 15% in 63% countries, whereas caesarean birth rates were lower than 10% in 28% countries [4]. Socioeconomic development, women’s education, urbanization, fertility, and physician availability are influencing the caesarean birth rates [4].

Concerns have been raised on overuse of caesarean delivery in all parts of the world. In China, the caesarean birth rate increased dramatically during the past three decades [5–10]. In 1988, the national caesarean birth rate was only 3.4%, but rapidly increased after 1990. Based on four National Health Service Surveys conducted in 1993, 1998, 2003, and 2008, the national caesarean birth rate increased from 14.9 to 64.1% in urban areas and from 1.5 to 23.6% in rural areas between 1993 and 2008 [11]. Moreover, at 46.2%, China was reported to have the highest caesarean birth rate in a World Health Organization global survey conducted in 2007–2008 [9].

Based on the evidence, national and local health commissions and medical professionals have been working on reducing caesarean birth rate especially nonmedically indicated caesarean birth in China. In the past few years, the government has undergone efforts to curtail the rising caesarean rate and promote vaginal deliveries by launching a number of different programs [10, 12]. The recent study reported the overall rate of caesarean birth in China was 34.9% in 2014 and there was a decline in some super cities from 2008 to 2014 [10], and a steadily declined trend of caesarean birth rate was also found between 2012 and 2016 reported in another study [12]. China is seemed to be the only country that has succeeded in reversing the rising trends in caesarean deliveries [12].

The relaxation of the one-child policy in November 2013 allows at least the only-child of either parent to have two children, and the introduction of the universal two-child policy in October 2015 allows each married couple to have two children [13]. The new policy might affect the caesarean rate by influencing women’s expectations and choices as scar uterus has been gradually identified as an important risk factor for following pregnancy [14]. For primiparas, vaginal birth was the primary choice of delivery mode. Women were aware of the consequences of caesarean birth for future pregnancies, and caesarean birth on maternal request was not as widely accepted. For multiparas with previous caesarean birth, repeat caesarean birth remains the norm [14].

However, few studies reported the caesarean birth rate was affected by the policy especially after the universal two-child policy period. The data regarding the caesarean birth rate change after the two-child policy are needed. The current large population-based study aimed to assess the prevalence of caesarean deliveries from 2013 to 2018 in China and to provide the latest information on caesarean birth rate changes after universal two-child policy era and further elaborate the possible reasons.

## Methods

### Study design and participants

This nationwide, retrospective, population-based study was based on National Free Pre-Pregnancy Check-ups Project (NFPCP). Supported by the National Health Commission and Ministry of Finance of the People’s Republic of China, NFPCP provides preconception care including free health examinations prior to pregnancy, risk assessments, consultations, and two rounds of follow-ups for pregnancy outcomes for reproductive-aged couples who wish to conceive in the near 6 months. The national project was initially set to promote reproductive health of rural resident couples and then gradually expanded to urban resident couples since 2013. Even after the expansion, the national project still mainly focused on the rural areas. Until 31 December 2018, the NFPCP have covered 3245 counties/districts across 31 provinces in mainland China. Detailed design, organization, and implementation of the NFPCP have described elsewhere (Additional file 4: Fig. S1) [15].

Briefly speaking, couples planning to conceive in the next 6 months were encouraged to participate in NFPCP at local maternal and child care service centres. Baseline information including age, education, residence address, ethnicity, medical history, and reproductive history were collected using a standardized questionnaire with a face-to-face interview, and medical examinations were conducted by trained healthcare staff. After the completion of the health examination, two rounds of follow-up interviews were then conducted by trained nurses to update pregnancy status and outcomes. The first-round follow-up was conducted via telephone within 3 months after the baseline examination. If the participants were not pregnant at the first interview, repeated inquiries were subsequently conducted within the next 3 months until 1 year after the baseline examination. The participants who became pregnant were contacted within 1 year after the early pregnancy follow-up to ascertain pregnancy outcomes. Delivery information for both mothers and newborns was obtained from the mother’s self-reported information, including delivery mode (caesarean, operative vaginal, or spontaneous vaginal birth), delivery date, foetal number, and birth weight.

This study was approved by the Institutional Research Review Board at the National Health Commission. Written informed consent was obtained from all NFPCP participants.

### Covariates

Body weight and height were measured with participants wearing light, indoor clothing, and no shoes. Body mass index (BMI) was calculated using weight/height^2^ (kg/m^2^). BMI < 18.5 was considered underweight, BMI ≥ 18.5 and < 24 were considered normal, BMI ≥ 24 and < 28 were considered overweight, and BMI ≥ 28 was considered obese according to the Chinese population standards [16]. Higher education level was defined as senior high school, college, or postgraduate. Primiparity and multiparity were defined as parity of 0 or ≥ 1, respectively. And parity here was the number of deliveries that occurred ≥ 28 weeks before the women participated in the NFPCP. Participants’ region was defined according to their geographical location, including Northeastern (Heilongjiang, Jilin, and Liaoning), Northern (Beijing, Tianjin, Shanxi, Inner Mongolia, and Hebei), Northwestern (Sinkiang, Qinghai, Gansu, Shaanxi, and Ningxia), Central (Hubei, Hunan, and Henan), Eastern (Shandong, Jiangsu, Shanghai, Zhejiang, Fujian, Anhui, and Jiangxi), Southern (Guangdong, Guangxi, and Hainan), and Southwestern (Tibet, Yunnan, Guizhou, Chongqing, and Sichuan). Multivariable adjusted odds ratios (OR) of covariates with caesarean birth were presented in Additional file 1: Table. S1.

### Statistical analysis

We used population-based individual data to estimate the yearly crude caesarean birth rate, and we used age structure from 1% national population sample survey in 2015 to standardize caesarean birth rate.

To examine the trend of caesarean birth rate over time, we used marginal effect of year to measure the expected change of caesarean birth rate on the conditional mean of covariates, which could minimize, or reduce at least, the negative effect caused by structural differences of annual participants’ characteristics. Marginal effect of year was calculated by the following formula stated as:

$$ P\left(Y=1|{\overline{X}}_{\left(\mathrm{year}\right)},\mathrm{year}=2013,2014,\dots, 2018\right)-P\left(Y=1|{\overline{X}}_{\left(\mathrm{year}\right)},\mathrm{year}=2016\right) $$

where *Y* is the caesarean birth, ${\overline{X}}_{\left(\mathrm{year}\right)}$ denotes the means (for continuous variables) and reference levels (for binary or categorical variables) of all the other variables in the model, and year is the year of caesarean birth. Year of 2016, the starting year of the universal two-child policy, was set as the reference year for comparing the trend of annual caesarean birth rate; reference level for covariates: age was ‘25–29 years’, BMI was ‘normal weight’, nationality was ‘Han nationality’, education was ‘no higher education’, household register type was ‘rural’, adverse pregnancy outcome history was ‘without adverse pregnancy outcomes’, parity was ‘primipara’, full-term births was ‘yes’, and number of foetus was ‘singleton’.

We also used interrupted time series (ITS) analysis to determine impacts of the universal two-child policy on the trend of caesarean birth. The following segmented regression model is used: *Y*_*t*_ = *β*_0_ + *β*_1_*T* + *β*_2_*X*_*t*_ + *β*_3_*TX*_*t*_, where *T* is the months elapsed since the start of this study, *X*_*t*_ is a dummy variable indicating the period before (coded as 0) after (coded 1) the implementation of policy (the implementation was set at November 2015), and *Y*_*t*_ is the standardized caesarean birth rate at time *t* or marginal effect of caesarean birth at time *t* (month in this study). And the parameter *β*_0_ represents the baseline level at *T* =0, *β*_1_ is interpreted as change of caesarean birth rate associated with a month increase before the implementation of policy, *β*_2_ is the rate change by the implementation at the short-term, and *β*_3_ indicates the rate change following the implementation in the long-term [17].

To explore the rural/urban or geographical difference of caesarean birth rate trend, we presented the crude rate, standardized rate, and marginal effects of year by participants’ residential registering type and regions of provinces, respectively. As the impacts of universal two-child policy on participants’ choice for delivery mode may be differ between primipara and multipara, we also conducted analyses according to parity. An additional analysis was also conducted by applying logistics regression to see whether baseline characteristics were associated with caesarean birth, and multivariable adjusted OR were used to measure magnitudes of their associations.

R software (version 3.2.2; https://www.r-project.org/) was used for all analyses. R package mfx (version 1.1) was used to estimate the marginal effect of delivery year. The *p* value < 0.05 was considered to be significant.

## Results

**Fig. 1:**
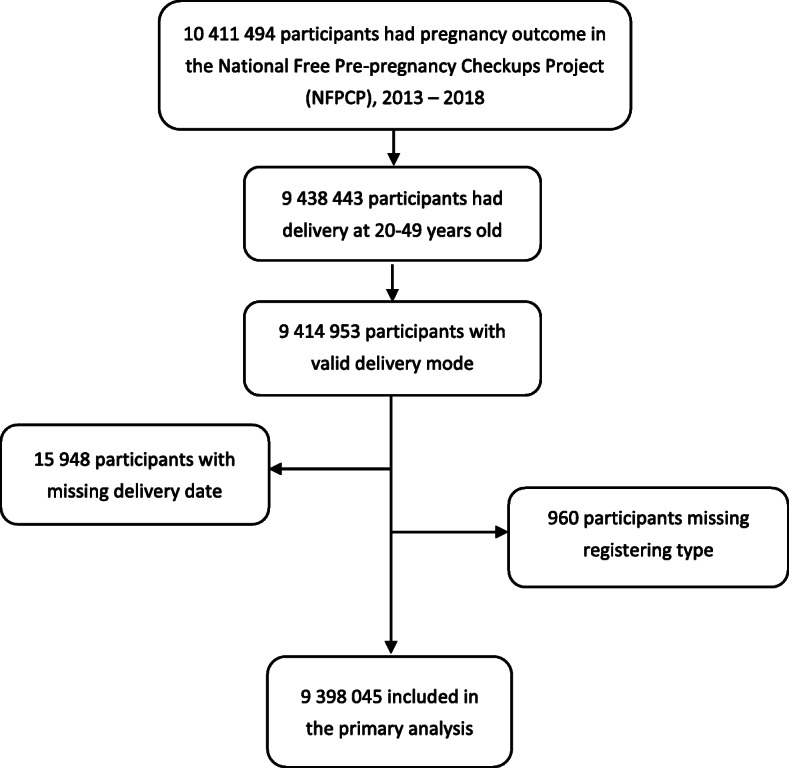
Flow diagram of included and excluded participants

NFPCP participants who completed deliveries between January 1, 2013, and December 31, 2018, were included in the current study. And among them, 9,438,443 participants were aged 20–49 years old at delivery. After sequentially excluding of participants with missing delivery mode, delivery date, and household registration type (rural or urban), 9,398,045 participants were included in the final analyses (Fig. 1).

Descriptive statistics were presented in Table 1. The proportion of participants with advanced maternal age (age ≥ 35 years) was 4.6% in 2013 and 11.8% in 2018. In 2013, 32.1% of the participants had at least a senior high school education compared with 46.4% in 2018. More obese (12.2% vs. 7.6%, *p* < 10^−5^) and overweight (14.6% vs. 12.4%, *p* < 10^−5^) participants were enrolled in 2018 compared to 2013. The proportion of primiparas decreased from 73.8% in 2013 to 48.7% in 2018. Then, we did a quantitative analysis measuring the association between covariates and caesarean birth. It was found that advanced maternal age [OR 1.79 (1.78–1.80)], higher education level [OR 1.16 (1.15–1.16)], overweight [OR 1.20 (1.20–1.21)] and obese women [OR 1.53 (1.52–1.54)], and complicated with adverse pregnancy outcomes in previous deliveries [OR 1.35 (1.35–1.36)] et al. were independent risk factors of caesarean birth (Additional file 1: Table. S1).

**Table 1:** Characteristics of the participants according to delivery year

Characteristics	2013	2014	2015	2016	2017	2018
***N***	1,360,384	1,813,753	1,552,952	1,706,939	1,709,637	1,254,380
**Age**						
20–	449,607 (33.1)	482,363 (26.6)	391,365 (25.2)	350,263 (20.5)	283,488 (16.6)	205,376 (16.4)
25–	648,870 (47.7)	948,058 (52.3)	818,991 (52.7)	884,704 (51.8)	770,323 (45.1)	563,423 (44.9)
30–	199,323 (14.7)	305,331 (16.8)	257,309 (16.6)	339,758 (19.9)	419,649 (24.6)	337,983 (26.9)
≥ 35	62,584 (4.6)	78,001 (4.3)	85,287 (5.5)	132,214 (7.8)	236,177 (13.8)	147,598 (11.8)
**Higher education**						
Yes	436,700 (32.1)	625,611 (34.5)	573,043 (36.9)	708,332 (41.5)	715,633 (41.9)	581,466 (46.4)
No	891,977 (65.6)	1,134,400 (62.5)	927,246 (59.7)	926,811 (54.3)	913,797 (53.5)	600,435 (47.9)
NA	31,707 (2.3)	53,742 (3.0)	52,663 (3.4)	71,796 (4.2)	80,207 (4.7)	72,479 (5.8)
**BMI**						
Underweight	191,759 (14.1)	267,236 (14.7)	227,708 (14.7)	232,675 (13.6)	209,234 (12.2)	159,800 (12.7)
Normal weight	876,038 (64.4)	1,147,895 (63.3)	979,551 (63.1)	1,051,466 (61.6)	1,026,015 (60.0)	750,469 (59.8)
Overweight	169,411 (12.4)	228,695 (12.6)	200,350 (12.9)	234,272 (13.7)	255,224 (14.9)	183,551 (14.6)
Obese	102,921 (7.6)	153,108 (8.4)	133,190 (8.6)	176,100 (10.3)	207,396 (12.1)	152,599 (12.2)
NA	20,255 (1.5)	16,819 (0.9)	12,153 (0.8)	12,426 (0.7)	11,768 (0.7)	7961 (0.6)
**Region**						
Northeast	17,087 (1.3)	21,362 (1.2)	12,880 (0.8)	17,295 (1.0)	16,405 (1.0)	13,631 (1.1)
North	70,111 (5.2)	132,151 (7.3)	83,421 (5.4)	113,796 (6.7)	99,505 (5.8)	72,659 (5.8)
Northwest	84,483 (6.2)	104,363 (5.8)	92,596 (6.0)	101,283 (5.9)	108,509 (6.4)	88,936 (7.1)
East	254,361 (18.7)	479,177 (26.4)	339,419 (21.9)	414,585 (24.3)	423,651 (24.8)	316,769 (25.3)
Central	554,208 (40.7)	657,323 (36.2)	637,957 (41.1)	656,494 (38.5)	625,204 (36.6)	441,871 (35.2)
South	250,558 (18.4)	275,153 (15.2)	253,124 (16.3)	260,102 (15.2)	268,554 (15.7)	180,011 (14.4)
Southwest	129,576 (9.5)	144,224 (8.0)	133,555 (8.6)	143,384 (8.4)	167,809 (9.8)	140,503 (11.2)
**Nationality**						
Han	1,242,454 (91.3)	1,662,843 (91.7)	1,412,724 (91.0)	1,562,677 (91.6)	1,556,050 (91.0)	1,129,277 (90.0)
Others	102,498 (7.5)	132,018 (7.3)	122,268 (7.9)	120,483 (7.1)	131,382 (7.7)	100,268 (8.0)
NA	15,432 (1.1)	18,892 (1.0)	17,960 (1.2)	23,779 (1.4)	22,205 (1.3)	24,835 (2.0)
**Adverse pregnancy outcomes in previous deliveries**						
Yes	224,699 (16.5)	298,746 (16.5)	241,003 (15.5)	284,002 (16.6)	337,703 (19.8)	253,686 (20.2)
No	1,124,249 (82.6)	1,505,067 (83.0)	1,302,460 (83.9)	1,414,285 (82.9)	1,363,499 (79.8)	994,880 (79.3)
NA	11,436 (0.8)	9940 (0.6)	9489 (0.6)	8652 (0.5)	8435 (0.5)	5814 (0.5)
**Parity**						
Primipara	1,004,419 (73.8)	1,254,131 (69.2)	1,036,872 (66.8)	1,026,506 (60.1)	800,238 (46.8)	610,610 (48.7)
Multipara	343,741 (25.3)	548,999 (30.3)	506,195 (32.6)	671,455 (39.3)	900,763 (52.7)	637,945 (50.9)
NA	12,224 (0.9)	10,623 (0.6)	9885 (0.6)	8978 (0.5)	8636 (0.5)	5825 (0.5)
**Full-term births**						
Yes	1,234,240 (90.7)	1,659,942 (91.5)	1,441,070 (92.8)	1,593,940 (93.4)	1,629,940 (95.3)	1,216,586 (97.0)
No	126,143 (9.3)	153,809 (8.5)	111,882 (7.2)	112,999 (6.6)	79,696 (4.7)	37,794 (3.0)
NA	1 (0)	2 (0)	0 (0)	0 (0)	1 (0)	0 (0)
**Number of foetus**						
Singleton	1,352,364 (99.4)	1,802,094 (99.4)	1,543,985 (99.4)	1,697,149 (99.4)	1,700,026 (99.4)	1,246,359 (99.4)
Multiple	8019 (0.6)	11,657 (0.6)	8967 (0.6)	9790 (0.6)	9602 (0.6)	8021 (0.6)
NA	1 (0)	2 (0)	0 (0)	0 (0)	9 (0)	0 (0)

Data are *n* (%). *NA* not available, *BMI* body mass index. BMI was calculated using weight/height^2^ (kg/m^2^). BMI < 18.5 was considered underweight, BMI ≥ 18.5 and < 24 were considered normal, BMI ≥ 24 and < 28 were considered overweight, and BMI ≥ 28 was considered obese according to the Chinese population standards. Higher education level was defined as senior high school, college, or postgraduate. Primiparity and multiparity were defined as parity of 0 and ≥ 1, respectively

**Fig. 2:**
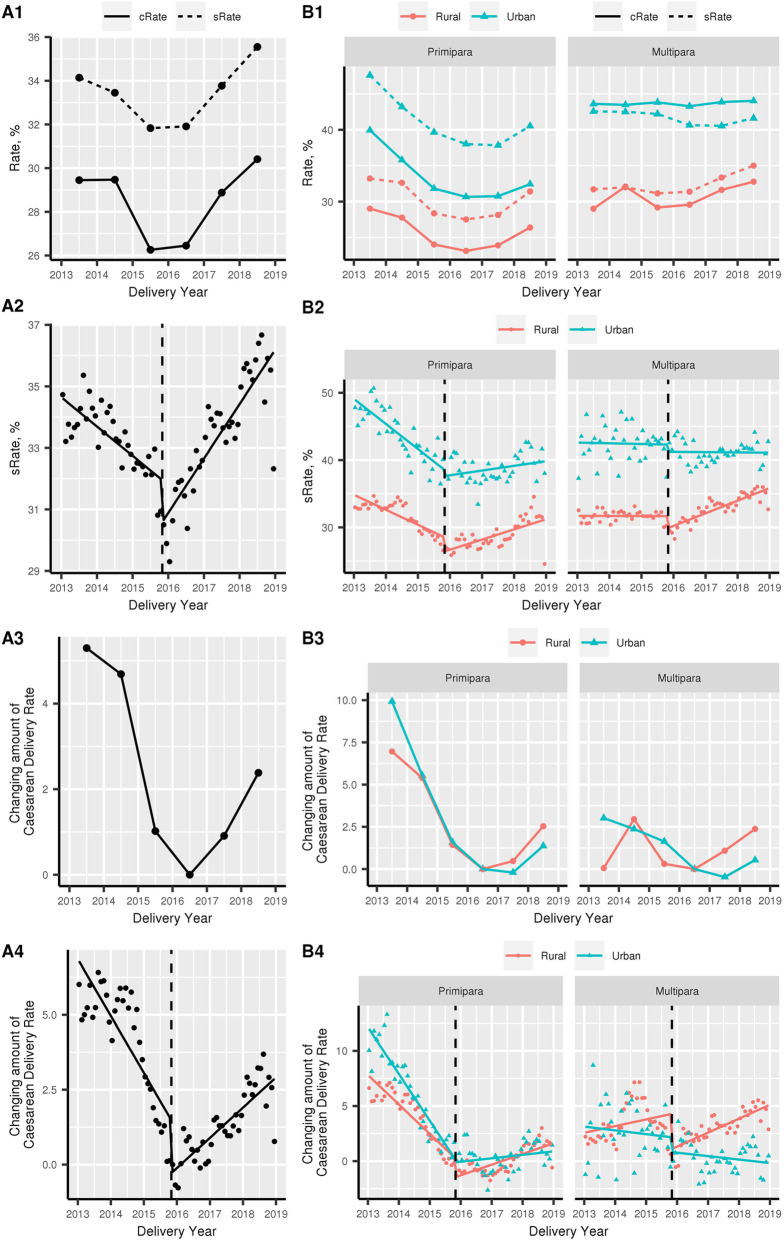
Caesarean birth rate of NFPCP participants in 2013–2018, before and after the universal two-child policy. **A1** Caesarean birth rate of NFPCP participants in 2013–2018; cRate, crude rate; sRate, standardized rate. **A3** After adjustment for baseline characteristics distributed differently in the study period, marginal effect results revealed the changing amount of caesarean birth rate. **A2**, **A4** Interrupted time series (ITS) analysis to determine impacts of the universal two-child policy on the trend of caesarean birth. **B1**–**B4** Trends of caesarean birth rate in different household register type (rural and urban) and parity (primipara and multipara)

In the current study, the standardized caesarean birth rate declined from 34.1% in 2013 to 31.8% in 2015 and increased to 35.6% in 2018 (Fig. 2A1 and Additional file 2: Table. S2). After adjustment for baseline characteristics distributed differently in the study period, marginal effect results revealed that the changing amount of caesarean birth rate decreased from 5.3% (95 confidence interval (CI) 5.2–5.4) in 2013 to 0.0% of the reference year 2016 and increased to 2.4% (95% CI 2.3–2.4) in 2018 (Fig. 2A3 and Additional file 2: Table. S2).

Next, we assessed the influences of universal two-child policy on the caesarean birth rate using ITS analyses. ITS results were consistent with the trend of caesarean birth rate (Fig. 2A2, A4, and Additional file 3: Table. S3). For standardized rate, ITS analysis showed the caesarean birth rate decreased by 0.1% (95% CI 0.1–0.1%) per month before the release of universal two-child policy, 1.3% (95% CI 0.6–2.1%) absolute drop during the policy release month, and increased by 0.2% (95% CI 0.1–0.2%) per month following the policy implementation. For marginal effects model, compared to the reference participants, caesarean birth rate decreased by 0.2% (95% CI 0.1–0.2%) for each month before the release of policy, 1.3% (95% CI 0.5–2.1%) absolute drop during the release month, and increased by 0.1% (95% CI 0.1–0.1%) per month following the implementation of policy (Fig. 2A2, A4, and Additional file 3: Table. S3).

Figure 2B1–B4 also presented trends of caesarean birth rate in different household register type and parity. For the study period before the release, the caesarean birth rate decreased by 0.2% (95% CI 0.2–0.3%) in rural primiparas and 0.2% (95% CI 0.1–0.2%) in urban primiparas and increased by 0.1% (95% CI 0.0–0.1%) in rural multiparas (Fig. 2B4 and Additional file 3: Table. S3). For the immediate change during the release month, participants with different household register type and parity showed an absolute decrease with minor differences. For the period after the policy release, the increasing trends were observed in rural participants and urban primiparas, but not in urban multiparas (Fig. 2B4 and Additional file 3: Table. S3)

**Fig. 3:**
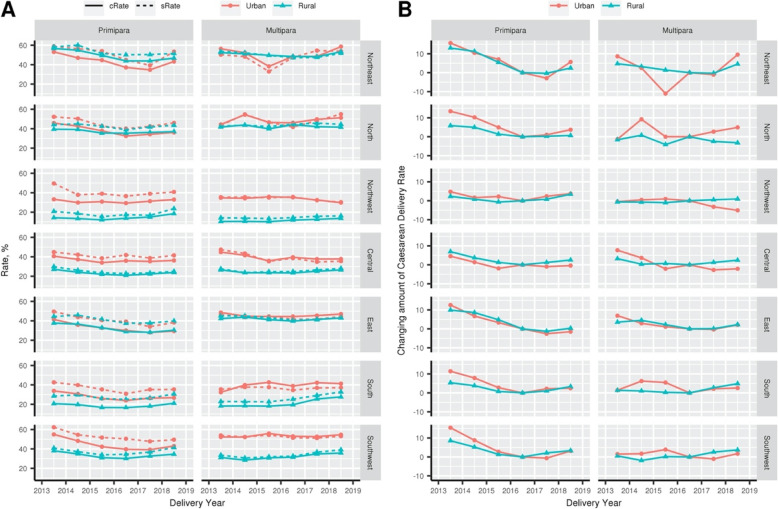
Caesarean birth rate of NFPCP participants in 2013–2018, according to participants region. **a** Caesarean birth rate of NFPCP participants in 2013–2018, according to participants region (cRate, crude rate; sRate, standardized rate). Participants’ region was defined according to their geographical location, including Northeastern, Northern, Northwestern, Central, Eastern, Southern, and Southwestern. **b** After adjustment for baseline characteristics distributed differently in the study period, marginal effect results revealed the changing amount of caesarean birth rate

The prevalence of caesarean birth rates within China varied regionally. In most parts of China, caesarean birth rates have a trend to decline from 2013 to 2016 and increases in 2017 and 2018. And the decreasing trend in primipara of all regions are more obvious than multipara (Fig. 3).

## Discussion

Using data from more than 9 million deliveries in 3245 counties/districts in China, we found that caesarean birth rates gradually declined from 34.1% in 2013 to 31.8% in 2015, consistent with recent reported overall decreased trend of caesarean birth rate in China between 2012 and 2016 [4]. In the current study, we also reported the caesarean birth rate has increased to 35.6% in 2018 after ‘universal two-child policy’.

To effectively control the increasing caesarean rate, especially caesarean deliveries without medical indications, the government has developed policies and measures, such as the use of the caesarean rate as an essential indicator to evaluate hospitals and promotion of vaginal deliveries through various programs. Health education and media publicity also help to instil confidence for spontaneous vaginal deliveries and promote understanding of maternal and neonatal safety. The Chinese Medical Association published clinical guidelines for caesarean birth, to reduce the occurrence of caesarean without medical indications. The new definition of ‘arrest of labour’ was applied in 2014 to further contribute to the reduction of caesarean birth [18].

The introduction of the two-child policy in China in October 2015 has led to more families having a second child. The planning second child would influence the choices around the first birth both for expected mothers and physicians. For primiparas, vaginal birth was the primary choice of delivery method. For multiparas with previous caesarean birth, repeat caesarean birth remains the primary choice [14]. The caesarean birth rate decline was observed during the initial universal two-child policy release, especially in primiparas both in urban and rural areas. The policy meanwhile led to an increase of the multiparous birth proportion, and the proportion in women with uterine scar doubled based on the recent data [12]. In the longer term after the policy release, it is not surprising that caesarean birth rate increased in multiparas because the women with uterine scar opt for elective caesarean deliveries.

It was noted that for the period after the policy release, the increasing trends of caesarean birth were also observed in primiparas both in rural and urban. To delve into the probable explanations of this circumstance, we further found that the proportions of population with risk factors including advanced maternal age, higher education level, overweight or obese women, and complicated with adverse pregnancy outcomes in previous deliveries and multiple pregnancy, which can significantly increase the risk of caesarean birth, have been increased year by year. As time went on, the influences of risk factors in the primiparas were beginning to outweigh the effect of the policy. This might partially explain the caesarean birth rate increase in the primiparas.

The next question is whether the increase of caesarean birth rate is due to the assumed ‘baby boom’ after the universal two-child policy. The relaxation of the one-child policy has been introduced gradually. The couples who were both only-child were permitted to have two children since 2007. The policy then allowed at least the only child of either parent to have two children since November 2013. Only 13.2% eligible couples applied for permission to have a second child by May 2015 [13]. Therefore, the initial relaxation of one-child policy did not lead to a baby boom. Actually, the birth rate increased from 12.07% in 2015 to 12.95% in 2016, 12.43% in 2017, and surprisingly decreased to 10.94% in 2018 (http://data.stats.gov.cn/easyquery.htm?cn=C01&zb=A0302&sj=2018). It seemed there was no baby boom after the universal two-child policy release as expected.

The optimal threshold of caesarean delivery use is hard to determine. It has been suggested caesarean delivery use reaches an optimal threshold ranging from 9 to 16% to improve maternal and neonatal survival [19]. China was thought to be the only country that has succeeded in reversing the rising trends in caesarean deliveries due to comprehensive strategies and various incentives in the past few years [12]. Although universal two-child policy has been implemented in China for several years till now, there is a lack of data regarding the contribution of the policy on the caesarean birth rate. A study recently reported that caesarean birth rates plateaued between 2012 and 2016 and the rates increased from 2016 to 2018 [20]. However, the study was limited by the use of crude rates without adjustment for maternal characteristics. Besides, the study did not report the underlying influencing factors. Another study showed that the launch of two-child policy did not alter the caesarean birth rate based on analysis of data from 2012 to 2016 using segmented logistic regression approach [21]. Nevertheless, the study assessed caesarean birth rate in two provinces and the conclusion could not be generalized. Besides, the study ended before the increase captured in the current study.

Our study provided more lines of evidence regarding the caesarean birth rate before and after ‘universal two-child policy’ and identified several high-risk factors (advanced maternal age, higher education level, overweight and obese women, complicated with adverse pregnancy outcomes in previous deliveries) for caesarean birth rate have been increased. In the current study, there is a noticeable increase of caesarean birth rate which is identified in 2017 and 2018, only 2–3 years after the ‘universal two-child policy’, although a significant but temporary decrease of caesarean birth rate appeared in 2016. It reminds us that the caesarean birth control is still a long process and all the strategies need to be continually reinforced both in primiparas and multiparas, such as promoting trial of labour after caesarean (TOLAC), performing external cephalic version for breech presentation, providing health education for childbearing women, and providing nutritional management during pregnancy to realize optimal maternal weight gain and avoid macrosomia.

There are several limitations to our analysis. One limitation is that we could not distinguish the indications for caesarean birth, such as foetal distress, cephalo-pelvic disproportion, previous uterine surgery, malpresentation, and suspected macrosomia [22–24]. Secondly, NFPCP study mainly targeted participants from rural areas in which the caesarean birth rate was relatively lower. Thus, we are not reporting the national caesarean birth rate here due to the sample representation. Nevertheless, the trends of caesarean birth rate changes in NFPCP study were consistent with the previous published national data [10, 12, 20].

## Conclusions

The decreasing trend of caesarean birth rate was reported after immediate release of the universal two-child policy. An increasing trend of caesarean birth rate was observed 2–3 years after the policy. It reminds us that the caesarean birth control is a long-lasting process and all the strategies need to be continually reinforced. Future studies are needed to further demonstrate the larger picture of caesarean birth rate changes influenced by the universal two-child policy in the long term.

## Supplementary information

**Additional file 1 : Table. S1**. Association between baseline characteristics and the risk of caesarean birth.

**Additional file 2 : Table. S2**. Marginal effects results revealed that the changing amount of caesarean birth rate.

**Additional file 3 : Table. S3**. Interrupted time series model to determine impacts of the universal two-child policy on the trend of caesarean birth.

**Additional file 4 : Figure S1**. Translated of detailed design, organization, and implementation of the NFPCP (described elsewhere *in Chinese*). [15].

## Data Availability

All relevant data are within the manuscript and its Supporting Information files.

## Abbreviations

ITS: Interrupted time series
NFPCP: National Free Pre-Pregnancy Check-ups Project
TOLAC: Trial of labour after caesarean
