# Supplementary material for: The trend of caesarean birth rate changes in China after ‘universal two-child policy’ era: a population-based study in 2013–2018
Source: BMC Med. 2020 Sep 15;18:249. doi: 10.1186/s12916-020-01714-7 (PMC7491061; doi:10.1186/s12916-020-01714-7)
Supplement: Supplementary file 1 — Additional file 1 : Table. S1. Association between baseline characteristics and the risk of caesarean birth. [file 12916_2020_1714_MOESM1_ESM.docx]

**Supplementary Table 1. Association between baseline characteristics and the risk of caesarean ~~delivery~~birth.**

| **Baseline Characteristics** | **No. of Caesarean deliveries (%)** | **OR** | **95% CI** |
| --- | --- | --- | --- |
| **Age** |  |  |  |
| 20- | 488556 (22.6) | 1.00 | Reference |
| 25- | 1254586 (27.1) | 1.17 | 1.17-1.18 |
| 30- | 631250 (34.0) | 1.49 | 1.49-1.50 |
| 35- | 295255 (39.8) | 1.79 | 1.78-1.80 |
| **Higher Education** |  |  |  |
| No | 1569191 (27.3) | 1.00 | Reference |
| Yes | 1100456 (30.2) | 1.16 | 1.15-1.16 |
| **Household type** |  |  |  |
| Urban | 228332 (36.9) | 1.00 | Reference |
| Rural | 2441315 (27.8) | 0.82 | 0.81-0.82 |
| **Nationality** |  |  |  |
| Others | 154838 (21.8) | 1.00 | Reference |
| Han | 2514809 (28.9) | 1.29 | 1.28-1.30 |
| **BMI** |  |  |  |
| Underweight | 309503 (24.0) | 0.91 | 0.90-0.91 |
| Normal weight | 1588948 (26.9) | 1.00 | Reference |
| Overweight | 409626 (32.2) | 1.20 | 1.20-1.21 |
| Obese | 361570 (39.1) | 1.53 | 1.52-1.54 |
| **Adverse Pregnancy Outcomes in Previous Deliveries** | | | |
| No | 2066628 (26.6) | 1.00 | Reference |
| Yes | 603019 (36.8) | 1.35 | 1.35-1.36 |
| **Parity** |  |  |  |
| Primipara | 1526123 (26.4) | 1.00 | Reference |
| Multipara | 1143524 (31.7) | 0.97 | 0.97-0.97 |
| **Full-term Births** |  |  |  |
| No | 186760 (30.0) | 1.00 | Reference |
| Yes | 2482887 (28.3) | 1.03 | 1.02-1.03 |
| **Number of Fetus** |  |  |  |
| Singleton | 2624119 (28.1) | 1.00 | Reference |
| Multiple | 45528 (81.2) | 11.14 | 10.90-11.40 |

Data are number of caesarean deliveries (rate of caesarean ~~delivery~~birth). BMI: body mass index. BMI was calculated using weight/height^2^ (kg/m^2^). BMI <18.5 was considered underweight, BMI ≥18.5 and <24 was considered normal, BMI ≥24 and <28 was considered overweight, and BMI ≥28 was considered obese according to the Chinese population standards. Higher education level was defined as senior high school, college, or postgraduate. Primiparity and multiparity were defined as parity of 0 or ≥1, respectively.
