# Supplementary material for: The trend of caesarean birth rate changes in China after ‘universal two-child policy’ era: a population-based study in 2013–2018
Source: BMC Med. 2020 Sep 15;18:249. doi: 10.1186/s12916-020-01714-7 (PMC7491061; doi:10.1186/s12916-020-01714-7)
Supplement: Supplementary file 2 — Additional file 2 : Table. S2. Marginal effects results revealed that the changing amount of caesarean birth rate. [file 12916_2020_1714_MOESM2_ESM.docx]

**Supplementary Table 2. Marginal effects results revealed that the changing amount of caesarean ~~delivery~~birth rate.**

| **Year** | **N** | **Crude Rate** | **Standardized Rate** | **Changing amount of Rate** | |
| --- | --- | --- | --- | --- | --- |
|  |  |  |  | **%** | **95% CI** |
| **Total Participants** | | | | | |
| **2013** | 400582 | 29.5 | 34.1 | 5.3 | 5.2-5.4 |
| **2014** | 534452 | 29.5 | 33.5 | 4.7 | 4.6-4.7 |
| **2015** | 407832 | 26.3 | 31.8 | 1.0 | 1.0-1.1 |
| **2016** | 451530 | 26.5 | 31.9 | 0.0 | Reference |
| **2017** | 493735 | 28.9 | 33.8 | 0.9 | 0.9-1.0 |
| **2018** | 381516 | 30.4 | 35.6 | 2.4 | 2.3-2.4 |
| **Urban Primipara** | | | | | |
| **2013** | 17673 | 39.9 | 47.6 | 9.9 | 9.6-10.2 |
| **2014** | 25653 | 35.8 | 43.2 | 5.5 | 5.3-5.8 |
| **2015** | 18898 | 31.8 | 39.7 | 1.6 | 1.3-1.8 |
| **2016** | 27832 | 30.7 | 38.0 | 0.0 | Reference |
| **2017** | 21531 | 30.8 | 37.8 | -0.2 | -0.4-0.0 |
| **2018** | 19473 | 32.4 | 40.5 | 1.4 | 1.1-1.6 |
| **Urban Multipara** | | | | | |
| **2013** | 1634 | 43.6 | 42.6 | 3.0 | 2.2-3.9 |
| **2014** | 3742 | 43.5 | 42.5 | 2.4 | 1.8-3.0 |
| **2015** | 10140 | 43.8 | 42.2 | 1.6 | 1.2-2.0 |
| **2016** | 19932 | 43.3 | 40.7 | 0.0 | Reference |
| **2017** | 36850 | 43.9 | 40.6 | -0.5 | -0.8-(-0.2) |
| **2018** | 23174 | 44.0 | 41.6 | 0.5 | 0.2-0.9 |
| **Rural Primipara** | | | | | |
| **2013** | 278601 | 29.0 | 33.2 | 7.0 | 6.9-7.0 |
| **2014** | 328365 | 27.8 | 32.6 | 5.4 | 5.3-5.5 |
| **2015** | 234885 | 24.0 | 28.4 | 1.4 | 1.4-1.5 |
| **2016** | 216409 | 23.1 | 27.5 | 0.0 | Reference |
| **2017** | 174577 | 23.9 | 28.1 | 0.5 | 0.4-0.5 |
| **2018** | 145292 | 26.4 | 31.4 | 2.5 | 2.5-2.6 |
| **Rural Multipara** | | | | | |
| **2013** | 98624 | 29.0 | 31.7 | 0.1 | 0.0-0.2 |
| **2014** | 173417 | 32.1 | 32.0 | 3.0 | 2.9-3.0 |
| **2015** | 140976 | 29.2 | 31.2 | 0.3 | 0.2-0.4 |
| **2016** | 184901 | 29.6 | 31.4 | 0.0 | Reference |
| **2017** | 258348 | 31.6 | 33.3 | 1.1 | 1.0-1.2 |
| **2018** | 191786 | 32.8 | 35.0 | 2.4 | 2.3-2.5 |

Data are number of caesarean deliveries and rate of caesarean ~~delivery~~birth (%). Marginal effects over year (taking 2016 as the reference year) were estimated by logistic regression using mfx package (ver. 1.1), holding age=25-29, BMI=Normal weight, higher education=no, nationality=Han, adverse pregnancy outcome=no, full term birth=yes, number of fetus=singleton.
