# Supplementary material for: The trend of caesarean birth rate changes in China after ‘universal two-child policy’ era: a population-based study in 2013–2018
Source: BMC Med. 2020 Sep 15;18:249. doi: 10.1186/s12916-020-01714-7 (PMC7491061; doi:10.1186/s12916-020-01714-7)
Supplement: Supplementary file 3 — Additional file 3 : Table. S3. Interrupted time series model to determine impacts of the universal two-child policy on the trend of caesarean birth. [file 12916_2020_1714_MOESM3_ESM.docx]

**Supplementary Table 3. Interrupted time series model to determine impacts of the universal two-child policy on the trend of caesarean ~~delivery~~birth.**

| **Model** | **Coefficients** | **Estimate** | **95% CI** | **Model** | **Coefficients** | **Estimate** | **95% CI** |
| --- | --- | --- | --- | --- | --- | --- | --- |
| **A2** |  |  |  | **A4** |  |  |  |
|  | β0 | 34.7 | 34.1-35.3 |  | β0 | 7.0 | 6.4-7.6 |
|  | β1 | -0.1 | -0.1-(-0.1) |  | β1 | -0.2 | -0.2-(-0.1) |
|  | β2 | -1.3 | -2.1-(-0.6) |  | β2 | -1.3 | -2.1-(-0.5) |
|  | β3 | 0.2 | 0.1-0.2 |  | β3 | 0.1 | 0.1-0.1 |
| **B2 – Rural Primipara** | | |  | **B4 – Rural Primipara** | | |  |
|  | β0 | 35.0 | 33.9-36.0 |  | β0 | 7.9 | 7.3-8.6 |
|  | β1 | -0.2 | -0.2-(-0.1) |  | β1 | -0.2 | -0.3-(-0.2) |
|  | β2 | -1.3 | -2.7-0.0 |  | β2 | -1.3 | -2.2-(-0.5) |
|  | β3 | 0.1 | 0.1-0.2 |  | β3 | 0.1 | 0.1-0.1 |
| **B2 – Rural Multipara** | | |  | **B4 – Rural Multipara** | | |  |
|  | β0 | 31.7 | 31.1-32.3 |  | β0 | 2.5 | 1.6-3.5 |
|  | β1 | 0.0 | 0.0-0.0 |  | β1 | 0.1 | 0.0-0.1 |
|  | β2 | -1.3 | -2.2-(-0.5) |  | β2 | -1.3 | -2.6-(-0.1) |
|  | β3 | 0.2 | 0.1-0.2 |  | β3 | 0.1 | 0.1-0.1 |
| **B2 – Urban Primipara** | | |  | **B4 – Urban Primipara** | | |  |
|  | β0 | 49.3 | 47.9-50.7 |  | β0 | 7.0 | 6.4-7.6 |
|  | β1 | -0.3 | -0.4-(-0.2) |  | β1 | -0.2 | -0.2-(-0.1) |
|  | β2 | -1.3 | -3.2-0.5 |  | β2 | -1.3 | -2.5-(-0.2) |
|  | β3 | 0.1 | 0.0-0.1 |  | β3 | 0.1 | 0.1-0.1 |
| **B2 – Urban Multipara** | | |  | **B4 – Urban Multipara** | | |  |
|  | β0 | 42.6 | 41.1-44.1 |  | β0 | 3.2 | 1.8-4.5 |
|  | β1 | 0.0 | -0.1-0.1 |  | β1 | 0.0 | -0.1-0.0 |
|  | β2 | -1.3 | -3.3-0.6 |  | β2 | -1.3 | -3.1-0.4 |
|  | β3 | 0.0 | -0.1-0.1 |  | β3 | 0.0 | -0.1-0.0 |

Data are rate of caesarean ~~delivery~~birth (%). Models A2, A4, B2, B4 were according to Figure 2. A2, A4: Interrupted time series (ITS) analysis to determine impacts of the universal two-child policy on the trend of caesarean ~~delivery~~birth. B2, B4 presented trends of caesarean ~~delivery~~birth rate in different household register type (rural and urban) and parity (primipara and multipara). The parameter β0 represents the baseline level at T=0, β1 is interpreted as change of caesarean ~~delivery~~birth rate per month before the implementation of policy, β2 is the rate change by the implementation at the short-term, and β3 is the rate change per month after the implementation at the long-term.
