# Supplementary material for: The trend of caesarean birth rate changes in China after ‘universal two-child policy’ era: a population-based study in 2013–2018
Source: BMC Med. 2020 Sep 15;18:249. doi: 10.1186/s12916-020-01714-7 (PMC7491061; doi:10.1186/s12916-020-01714-7)
Supplement: Supplementary file 4 — Additional file 4 : Figure S1. Translated of detailed design, organization, and implementation of the NFPCP (described elsewhere in Chinese). [15]. [file 12916_2020_1714_MOESM4_ESM.docx]

**Supplementary Figure 1.**

Translated of detailed design, organization, and implementation of the NFPCP (described elsewhere *in Chinese*). [Zhang S, Wang Q, Shen H. Design of the national free proception health examination project in China. Zhonghua yi xue za zhi. 2015;95(3):162-5.]
